# Does a postmortem redistribution affect the concentrations of the 7 azaindole-derived synthetic cannabinoid 5F-MDMB-P7AICA in tissues and body fluids following pulmonary administration to pigs?

**DOI:** 10.1007/s00204-024-03815-1

**Published:** 2024-07-02

**Authors:** Adrian A. Doerr, Frederike Nordmeier, Nadja Walle, Matthias W. Laschke, Michael D. Menger, Markus R. Meyer, Peter H. Schmidt, Nadine Schaefer

**Affiliations:** 1 Institute of Legal Medicine, Saarland University, Building 49.1, 66421 Homburg, Germany; 2 Institute of Legal Medicine, University Hospital Schleswig-Holstein, Building U35, 24105 Kiel, Germany; 3 Institute for Clinical & Experimental Surgery, Saarland University, Building 65/66, 66421 Homburg, Germany; 4 Department of Experimental and Clinical Toxicology, Center for Molecular Signaling (PZMS), Saarland University, Building 46, 66421 Homburg, Germany

**Keywords:** Synthetic cannabinoids, 5F-MDMB-P7AICA, 7-Azaindole, Pigs, Postmortem redistribution

## Abstract

Many fatal intoxications have been reported in connection with the consumption of newer, highly potent synthetic cannabinoids. Yet, a possible postmortem redistribution (PMR) might complicate reliable interpretation of analytical results. Thus, it is necessary to investigate the PMR-potential of new synthetic cannabinoids. The pig model has already proven to be suitable for this purpose. Hence, the aim of this study was to study the PMR of the synthetic cannabinoid 5F-MDMB-P7AICA and its main metabolite 5F-MDMB-P7AICA-dimethylbutanoic acid (DBA). 5F-MDMB-P7AICA (200 µg/kg body weight) was administered by inhalation to anesthetized and ventilated pigs. At the end of the experiment, the animals were euthanized and stored at room temperature for 3 days. Tissue and body fluid samples were taken daily. Specimens were analyzed after solid phase extraction using a standard addition method and LC–MS/MS, blood was quantified after protein precipitation using a validated method. In perimortem samples, 5F-MDMB-P7AICA was found mainly in adipose tissue, bile fluid, and duodenum contents. Small amounts of 5F-MDMB-P7AICA were found in blood, muscle, brain, liver, and lung. High concentrations of DBA were found primarily in bile fluid, duodenum contents, urine, and kidney/perirenal fat tissue. In the remaining tissues, rather low amounts could be found. In comparison to older synthetic cannabinoids, PMR of 5F-MDMB-P7AICA was less pronounced. Concentrations in blood also appear to remain relatively stable at a low level postmortem. Muscle, kidney, fat, and duodenum content are suitable alternative matrices for the detection of 5F-MDMB-P7AICA and DBA, if blood specimens are not available. In conclusion, concentrations of 5F-MDMB-P7AICA and its main metabolite DBA are not relevantly affected by PMR.

**Supplementary Information:**

The online version contains supplementary material available at 10.1007/s00204-024-03815-1.

## Introduction

Initially a “legal alternative” to classical drugs of abuse, trading or even possession of most new psychoactive substances (NPS) has become illegal in many countries due to extensive legal restrictions. However, these legal novelties probably were the reason for a decrease of seizures over the last years, but could not prevent an ongoing use and development of new substances. Synthetic cannabinoids still represent the highest number of NPS reported for the first time to the EU Early Warning System (EMCDDA 2023). In 2022 alone, 41 NPS were reported for the first time with 24 of those being synthetic cannabinoids (EMCDDA 2023).

One serious issue that makes these substances so dangerous is that there are no pharmacological safety studies. The consumer acts as a guinea pig, so to speak, as the potencies of novel cannabinoids are still unknown at the beginning. Highly potent synthetic cannabinoids are still leading to serious and even fatal intoxications (Bo et al. 2024; Alzu’bi et al. 2024; de Oliveira et al. 2023) after smoking/inhaling, which is the common route of administration (Xu et al. 2024). A recent accumulation of death cases in Hungary was related to the intake of methyl 2-({[1-(4-fluorobutyl)-1*H*-indol-3-yl]carbonyl}amino)-3,3-dimethylbutanoate (4F-MDMB-BICA) (De Morais et al. 2020). These trends underline the persistent relevance of research regarding the toxicokinetics (TK) of synthetic cannabinoids, especially those with a methyl-dimethyl-butanoic acid (MDMB) structure, contained by many newer synthetic cannabinoids. A carboxamide structure element showed a rapid ester cleavage often leading to only little or non-measurable concentrations of the parent compound in blood or urine samples of users (Adamowicz et al. 2019; Krotulski et al. 2020; Yeter and Erol Ozturk 2019).

This phenomenon was also reported in a rather recent death case with a prolonged survival time after ingestion of the 7-azaindole derived synthetic cannabinoid (SC) methyl[2-[1-(5-fluoropentyl)-1*H*-pyrrolo[2,3-b]pyridin-3-yl]formamido]-3,3-dimethylbutanoate (5F-MDMB-P7AICA), with only low amounts of the parent compound being found compared to relatively high concentrations of the dimethylbutanoic acid (DBA) metabolite (Walle et al. 2023).

Hence, investigating the metabolism and finding potential analytical targets even regarding PM toxicology, are important research issues. Besides in vitro studies using human liver microsomes, human hepatocytes or zebrafish larvae, one possibility is the analysis of authentic case material, e.g., in the framework of a potential poisoning. (Presley et al. 2020; Gaunitz et al. 2018).

5F-MDMB-P7AICA is also known as 7′N-5F-ADB or MDMB-5F-P7AICA and represents a structural isomer of 5F-MDMB-PINACA (also known as 5F-ADB), having been responsible for a number of intoxications and death cases over the last years (Barcelo et al. 2017; Yeter and Erol Ozturk 2019). Synthetic cannabinoids with a 7-azaindole core structure seem to be more stable compared to azaindole or indole-core synthetic cannabinoids regarding metabolism and storage degradation (Krotulski et al. 2020; Walle et al. 2021), nevertheless, an extensive metabolic ester cleavage of the MDMB structure was also observed (Doerr et al. 2020). Postmortem redistribution (PMR) of drugs further complicates the assessment of blood concentrations in fatal cases. Depending on the analytes’ properties, its respective amount at the time of death and the postmortem interval (PMI), a PMR e.g., from sites of higher concentration to sites of lower concentration in the deceased body leads to altered concentrations as compared to those at the time of death. These changes might entail wrong conclusions concerning the lethal impact (Skopp 2012).

In a review of 74 authentic death case studies, Giorgetti et al. (2020) tried to assess a possible PMR of synthetic cannabinoids. However, they were not able to draw general conclusions, as only few data were available regarding the tissue distribution. Depending on the SC structure and the respective case, a PMR had been assumed. For example, a quotient of central/peripheral blood (C/P ratio) near 1 was found in a case of MDMB-CHMICA 12 h after death, which is not indicative of PMR (Gaunitz et al. 2018). Yet, central blood (CB) levels significantly exceeded peripheral blood (PB) levels in the case of Zaitsu et al. (2015) for MAM-2201, AM-1220, and AM-2232 (PMI: 20 h). As a conclusion, C/P ratios might lay above 1, if the time interval between smoking and death is quite short. The explanation for this phenomenon might be the high drug concentration in the lungs right after consumption. Following the concentration gradient, the consumed SC is released to surrounding vessels and tissues, especially to the left ventricle (Moriya and Hashimoto 1999).

These case reports, although providing insights from authentic scenarios, bear the imponderability of individual uncontrolled settings with mostly unknown time and dose of consumption and frequent mixed consumption of synthetic cannabinoids and various drugs. In addition, the often unknown PMR might further complicate the interpretation of the analytical data.

To overcome this bias, systematic and controlled studies are inevitable. However, such studies are not feasible in humans. The pig model has been shown to be suited in terms of TK studies for several pharmaceuticals and drugs over the last years, mostly due to the anatomical and metabolic similarities. In this context, we established a sophisticated pig model for the elucidation of TK models for tetrahydrocannabinol as well as the synthetic cannabinoids JWH-210 and RCS-4 (Schaefer et al. 2019, 2020a). On the basis of the THC pig data, we were able to predict human exposure applying our model to data from literature.

Regarding metabolism, a high similarity, as compared to human metabolism, has already been shown in an earlier study for 5F-MDMB-P7AICA (Doerr et al. 2020). Furthermore, the potential of relatively high sample volumes favors a study design with repeated sample drawings even PM.

For these reasons, in the present study different tissues and body fluids were sampled from pigs several hours after pulmonary administration of 5F-MDMB-P7AICA and analyzed to determine the concentration of the parent compound and its DBA metabolite to identify the perimortem distribution pattern (PMI 0). Subsequently, the postmortem concentrations were obtained by repeated daily sampling of the matrices over three days (PMI 1–3) to assess, whether a possible PMR of the parent compound and its main metabolite could be observed.

## Materials and methods

### Chemicals and reagents

HPLC grade acetonitrile, ethanol absolute, methanol p.a., acetone p.a., and HPLC grade water were purchased from Fisher Scientific (Loughborough, United Kingdom). Di-potassium hydrogen phosphate, acetic acid (100%), formic acid (98–100%), aqueous sodium hydroxide solution (1 M) and β-glucuronidase/aryl sulfatase from *Helix pomatia* were obtained from Merck (Darmstadt, Germany). 5F-MDMB-P7AICA DBA (1 mg in 100 µl acetonitrile) and AB-FUBINACA-d4 (1 mg/mL in methanol) were purchased from LGC Standards (Wesel, Germany). 5F-MDMB-P7AICA (1 mg) was obtained from Cayman Chemical (Ann Arbor, USA). Furthermore, a larger amount of 5F-MDMB-P7AICA (~ 1 g, 80% purity, 20% non-toxic degradation products) was purchased as ‘research chemical’ from an internet provider (www.buyresearchchemicals.de) falsely labelled by the vendor as 4′N-5F-ADB (Richter et al. 2019). Molecular formula, CAS number, SMILES ID and InChi code of 5F-MDMB-P7AICA, DBA metabolite and AB-FUBINACA-d4 each are listed in Supplementary Table 1.

The buffers were prepared as described in a previous study (Schaefer et al. 2015). Briefly, for the phosphate buffer (pH 9, 0.1 M) 22.82 g di-potassium hydrogen phosphate was dissolved in 1 L of deionized water. The acetate buffer (pH 4, 0.1 M) was prepared by diluting 5.7 mL anhydrous acetic acid and 16 mL aqueous sodium hydroxide solution (1 M) in 1 L deionized water.

### Calibrators used for the standard addition approach

For preparation of standard stock solutions of 5F-MDMB-P7AICA (1 mg/mL), 5 mg solid substance were dissolved with 5 mL ethanol. To generate working solutions the standard stocks (5F-MDMB-P7AICA) or the liquid standard references (DBA) were diluted with ethanol. The concentrations of the calibrators used for standard addition are listed in Table 1.

**Table 1:** Calibrator concentrations of 5F-MDMB-P7AICA and its dimethyl butanoic acid (DBA) metabolite used for the standard addition approach divided between the different approaches as well as the various specimens in ng/g tissue/ body fluid specimen

Specimen	Calibrator conc. of 5F-MDMB-P7AICA [ng/g]			Calibrator conc. of DBA [ng/g]		
	1	2	3	1	2	3
Perimortem specimens						
Brain/lung/liver/muscle tissue	0.5	1.0	1.5	0.5	1	1.5
Kidney	0.5	1.0	1.5	5	10	15
Urine	n.a	n.a	n.a	40	80	120
Duodenum content	2	4	6	50	100	150
Bile fluid	2 or 10	4 or 20	6 or 30	10 or 100	20 or 200	30 or 300
Fat	20	40	60	1	2	3
Postmortem specimens						
Brain/lung/liver muscle tissue	0.5	1.0	1.5	0.5	1.0	1.5
Liver	0.5	1.0	1.5	1	3	5
Kidney	0.5	1.0	1.5	5	10	15
Duodenum content	0.5	1.0	1.5	2	4	6
Bile fluid	5	10	15	10 or 100	20 or 200	30 or 300
Fat	20	40	60	1	2	3

*n.a.*  not added

All solutions were stored at − 20 °C.

### Animals

The experiments were conducted in compliance with the German legislation on protection of animals and the National Institutes of Health Guide for the Care and Use of Laboratory Animals (permission number 32/2018). Six domestic male pigs (Swabian Hall strain; body weight [BW] 40–51.2 kg, 3 months old) were kept with free access to water and standard daily food (OlymPig fattening feed, Raiffeisen, Münster, Germany). One night prior to the experiments, the animals were kept fasting. The animals had a dark/light cycle of 12 h. The room temperature was 22 ± 1 °C with a humidity of 55 ± 10%.

### Surgical procedures

Surgical procedures were performed as described elsewhere (Doerr et al. 2020; Schaefer et al. 2017, 2019, 2020a) for anesthesia, ventilation, intravital collection of specimens and surveillance of vital parameters. Details are listed in the Supplementary Material. Vital parameters at the time of death: blood pressure, pulse, rectal temperature and O_2_-saturation are depicted in Supplementary Table 3.

### Study design

As previously described (Doerr et al. 2020; Schaefer et al. 2019, 2020a), an ethanolic solution of 5 mg/mL 5F-MDMB-P7AICA was prepared. An aliquot of 1.600–2.048 mL was filled up with ethanol to a total volume of 2 mL, if needed to achieve a concentration of 200 µg per kg BW. The SC was administered inhalatively over 6.5–8 min, using a M-Neb flow + ventilation ultrasonic nebulizer MN-300/7 (Nebutec, Elsenfeld, Germany) in the inspiration-triggered mode.

Animal euthanasia was conducted eight hours after the drug administration using T 61 (embutramide, 0.12 mL/kg BW, Intervet Deutschland GmbH, Unterschleißheim, Germany). Afterwards, the abdominal cavity was opened. Samples of the following organs, tissues and body fluids were collected (PMI 0) and stored at − 20 °C until further analysis: Brain (cerebrum), lungs and liver with no differentiation between the lobes, kidneys, muscle tissue (from the hindleg), adipose tissue (subcutaneous (sc), dorsal, perirenal), bile fluid, duodenum content, urine (only at PMI 0) and PB (V. jugularis) as well as CB.

The abdominal cavity was sutured leaving the organs in situ and the animal bodies were kept at room temperature in a supine position. Analogously, samples were taken again after 24, 48 and 72 h (PMI 1–3), respectively. Yet, PM PB specimens were obtained by sampling the coagulated blood from the V. femoralis or V. brachialis. For this purpose, the whole vessel was sampled and the blood was drawn therefrom using a pipette with a wide lumen.

### Sample preparation

#### Tissue specimens and body fluids

Specimens were prepared according to a previous published method (Schaefer et al. 2017, 2019, 2020a) with changes regarding the applied buffers and the amount of acetonitrile. An amount of 2 g of solid tissue (brain, lung, liver, kidney and muscle tissue) was homogenized (1:5 w/w with water), respectively and 1 g of body fluids (bile fluid, duodenum content, and urine) was diluted (1:10 w/w for bile and duodenum content, 1:5 w/w for urine, respectively, with water). The samples were stored at − 20 °C.

To determine the standard addition calibration curves, four 0.5 g aliquots were added to 20 µL of an ethanolic stable-isotope-labeled internal standard solution (SIL-IS, 1 ng/20 µL AB-FUBINACA-d4) and 25 µL of ethanol or an ethanolic solution of the analytes.

Subsequently, the solution was mixed with 500 µL of acetate buffer and 50 µL of β-glucuronidase/arylsulfatase and incubated for 2 h at 60 °C to induce enzymatic hydrolysis of the glucuronidated DBA (phase-II metabolite).

For the following protein precipitation, the samples were mixed with 500 µL of acetonitrile and centrifuged at 3500*g* for 8 min. The supernatants were transferred to 1 mL phosphate buffer (pH 9) vortexed and centrifuged at 3500*g* for 8 min again.

Solid phase extraction (SPE) was carried out using Strata C18 end capped cartridges (Phenomenex, Aschaffenburg, Germany), previously conditioned with 2 × 3 mL methanol and 3 mL phosphate buffer. After loading the samples, the columns were washed with 3 mL phosphate buffer, 3 mL acetic acid (0.25 M) and 3 mL deionized water, respectively. 60 μL acetone was added and columns were dried for 5 min using negative pressure (about 33 kPa). Thereafter, the analytes were eluted with a mixture of 1.5 mL methanol-acetone (1:1, v/v) and the eluate was evaporated under a gentle stream of nitrogen at 60 °C. The dry residues were resuspended in 100 μL of a 1:1 (v/v) mixture of mobile phases A (0.1% aqueous formic acid) and B (0.1% formic acid in acetonitrile). 20 µL were injected for the analysis into the liquid-chromatography tandem-mass-spectrometry (LC–MS/MS) system.

#### Blood specimens

As the small amount of matrix did not allow for a standard addition method in PB, a previously validated method was applied for these samples (recovery ~ 75% and more, no relevant matrix effects, linear calibration with a weighting factor of 1/*x*^2^ for parent and 1/*x* for metabolite, calibration range 0.5 ng/mL-50 ng/mL (both analytes), limit of detection 0.05 ng/mL (both analytes), lower limit of quantification 0.5 ng/mL) (Walle et al. 2021). Briefly, 20 µL of a SIL-IS solution was mixed with 25 µL ethanol, 50 µL water and 50 µL blood. Precipitation was performed by adding 500 µL of acetonitrile and shaking for about 5 min. After centrifugation for 5 min at 12,000*g*, the supernatants were transferred to a new vial and gently evaporated under a nitrogen flow at 60 °C. The residues were reconstituted in 50 µL of a mixture of mobile phases A and B (1:1, v/v) and 20 µL were injected onto the LC–MS/MS system. The concentrations were quantified by a calibration.

### Standard addition method

5F-MDMB-P7AICA and DBA were quantified in tissue and body fluid samples using the standard addition method. To determine the calibration curves, four 0.5 g aliquots were prepared: one native and three with addition of differently concentrated standard mixtures consisting of the two analytes (see Table 1). The analyte/SIL-IS area ratio was plotted against the calibrator concentration. The regression equations could be determined from the curves by the term *y* = *a* *x* + *b*. Calculation was performed using Microsoft Office Excel 2016 (Redmond, WA, USA). The unknown concentration corresponds to the intersection point of the axis of abscissa and results from the slope (*a*) and the point of intersection with the axis of ordinate (*b*) (Schaefer et al. 2020a).

### Apparatus

LC–MS/MS conditions including the chromatographic, instrumentation, and mass spectrometric conditions were identical to a recently published study (Walle et al. 2021) and are listed in detail in the Supplementary material and Supplementary Table 2.

### Statistical tests

For the evaluation of concentration changes over the time of observation, a non-parametric Friedman-test (*p* < 0.05) followed by a Dunn’s multiple comparison post hoc test was applied for each matrix. Calculations were performed using GraphPad Prism 9.0.1 (GraphPad Software, San Diego, CA, USA).

## Results and discussion

### Route of administration

Synthetic cannabinoids are mostly consumed by inhalation, e.g. by smoking of herbal mixtures spiked with the substances or by heating the drug on a metal plate. In addition, so-called C-liquids containing synthetic cannabinoids are commonly vaporized (Xu et al. 2024). For this reason, we administered the SC via inhalation using a nebulizer in the inspiration-triggered mode. In contrast to a permanent nebulization, the triggered mode allowed for successive nebulization (< 0.2 mL/min) of the drug solution synchronized with each inspiratory phase. This procedure enabled to mimic an authentic consumption scenario.

### Method development

#### Tissues and body fluids

Extraction was applied according to a method used to quantify cannabinoids in a previous study (Schaefer et al. 2019). Because the extraction efficiency of the DBA was deemed too low, the method had to be optimized. Regarding the precipitation step, 1 mL acetonitrile as used in the earlier study led to acceptable amounts of parent substance in the extract. However, only low amounts of DBA could be retrieved. A reason might be a lower retention on the extraction cartridges due to the free carboxylic acid. A reduced volume of only 0.5 mL acetonitrile enhanced the amount of DBA found in the extract considerably. Replacement of sodium carbonate solution with a di-potassium hydrogen phosphate solution in the next step further enhanced the amount of analytes. An additional centrifugation step prior to loading the samples onto the cartridges appeared to be helpful to increase the flow during SPE.

Quantification in tissue samples was performed using the standard addition approach. This method bears the disadvantage of multiple analyses per sample. Even if it is more labor-intensive as compared to the conventional method validation, the approach is recommended for postmortem samples due to the possible great variations in matrix composition that would render an external calibration practically useless (Peters et al. 2007). Using this specific approach, matrix-matched calibration curves are applied. Furthermore, common validation procedures require the usage of blank matrix from different individuals for the assessment of several parameters. However, in case of PM specimens this might lead to unrepresentative results, as interindividual biological variances of the same matrix samples have to be considered. For this purpose, national and international guidelines recommend the application of the standard addition method for quantification of drugs in (PM) tissue specimens (GTFCh 2018; Jickells and Negrusz 2008; Peters et al. 2007; Skopp 2010; SOFT/AAFS 2006).

The regression coefficients (*r*^2^) were consistently > 0.9, guaranteeing adequate quantification.

### Statistical tests

We chose the Friedman test, because it can be used when the requirements for a parametric method are not met. Non-parametric methods are also known as ‘unconditional methods’, as they place fewer requirements on the distribution of the measured values in the population. For example, the data does not have to be normally distributed and the dependent variable only has to be ordinal scaled. A Friedman test can also be calculated for small samples and outliers. As we observed huge variations, even between specimens of the same individual, we assumed that the results were not normally distributed. The Dunn’s post-hoc test performs pairwise comparisons between each independent group and provides information, which groups differ statistically significant at some level.

### Perimortem concentrations and distribution patterns

**Fig. 1:**
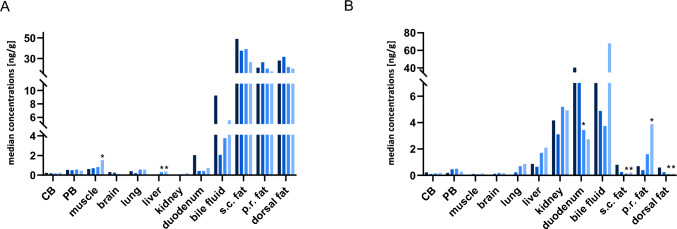
Median perimortem and postmortem concentrations of **A** MDMB-P7AICA and **B** MDMB-P7AICA-dimethylbutanoic acid metabolite in pig tissue and body fluid specimens following pulmonary administration of 200 µg/kg body weight (*n* = 6) of MDMB-P7AICA. ; *CB* central blood, *PB* peripheral blood, *s.c.* subcutaneous, *p.r.*  perirenal; *Statistical significant difference (*p* < 0.05)

The mean concentrations and their standard deviation (SD) of the parent and DBA calculated in specimens collected at PMI 0 are listed in Table 2. The median concentrations are depicted in Fig. 1A and B. Highest concentrations of the parent compound were found in the fat tissues and duodenum content as well as in bile fluid. Small amounts of 5F-MDMB-P7AICA were found in CB, PB, muscle, brain, liver and lung. No parent compound could be found in urine and kidney. This finding is not quite surprising, as this organ is affected with excretion processes. Thus, rather more hydrophilic metabolites are supposed to be found in this tissue.

**Table 2:** Mean concentrations ± standard deviations (SD) of 5F-MDMB-P7AICA and its dimethyl-butanoic acid metabolite (DBA) in ng/mL or ng/g measured in different tissue and body fluid specimens collected 8 h after inhalative drug administration (postmortem interval = PMI 0) as well as 24 h (PMI 1), 48 h (PMI 2), and 72 h (PMI 3) after euthanasia of six pigs and a following storage at room temperature

	PMI 0	PMI 1	PMI 2	PMI 3
*5F-MDMB-P7AICA*				
PB	0.57 ± 0.19 *n* = 6	0.67 ± 0.37 *n* = 6	0.58 ± 0.35 *n* = 6	0.43 ± 0.16 *n* = 6
CB	0.21 ± 0.10 *n* = 6	0.25 ± 0.10 *n* = 5	0.20 ± 0.090 *n* = 5	0.24 ± 0.11 *n* = 5
Urine	neg	n.a	n.a	n.a
Lung	0.41 ± 0.26 *n* = 6	0.50 ± 0.085 *n* = 3	0.65 ± 0.37 *n* = 5	0.71 ± 0.54 *n* = 5
Liver	0.16 ± 0.043 *n* = 3	0.21–0.29 *n* = 2	1.1 ± 2.0 *n* = 6	0.44 ± 0.18 *n* = 5
Kidney	neg	0.090–0.10 *n* = 2	0.17 ± 0.16 *n* = 5	0.23 ± 0.088 *n* = 5
Brain	0.33 ± 0.15 *n* = 6	0.23 ± 0.10 *n* = 6	0.10 ± 0.065 *n* = 6	0.16 ± 0.16 *n* = 4
Muscle	0.62 ± 0.10 *n* = 6	0.77 ± 0.28 *n* = 6	1.0 ± 0.67 *n* = 6	2.3 ± 2.1 *n* = 6
Dorsal fat	30 ± 16 *n* = 6	35 ± 26 *n* = 6	25 ± 15 *n* = 6	33 ± 27 *n* = 6
s.c. fat	51 ± 30 *n* = 6	53 ± 43 *n* = 6	40 ± 28 *n* = 6	35 ± 26 *n* = 6
Perirenal fat	33 ± 31 *n* = 6	30 ± 20 *n* = 6	32 ± 35 *n* = 6	22 ± 19 *n* = 6
Bile fluid	11 ± 9.9 *n* = 6	3.5 ± 3.1 *n* = 6	3–7-8.6 *n* = 3^1^	3.6–7.4 *n* = 2^2^
Duodenum content	2.7 ± 3.1 *n* = 6	0.44 ± 0.22 *n* = 6	0.59 ± 0.48 *n* = 5	0.89 ± 0.71 *n* = 6
*DBA*				
PB	0.18 ± 0.11 *n* = 6	0.47 ± 0.21 *n* = 6	0.48 ± 0.16 *n* = 6	0.28 ± 0.093 *n* = 6
CB	0.22 ± 0.11 *n* = 6	0.32 ± 0.40 *n* = 5	0.21 ± 0.17 *n* = 5	0.20 ± 0.10 *n* = 5
Urine	78 ± 45 *n* = 6	n.a	n.a	n.a
Lung	0.20 ± 0.071 *n* = 4	0.33 ± 0.21 *n* = 5	0.92 ± 0.69 *n* = 6	0.89 ± 0.57 *n* = 5
Liver	0.87 ± 0.37 *n* = 6	1.2 ± 1.4 *n* = 6	1.8 ± 1.2 *n* = 6	2.8 ± 1.9 *n* = 6
Kidney	5.4 ± 4.7 *n* = 6	5.1 ± 5.0 *n* = 6	7.0 ± 5.9 *n* = 6	6.5 ± 5.9 *n* = 6
Brain	neg	0.13 ± 0.056 *n* = 6	0.18 ± 0.065 *n* = 6	0.16 ± 0.043 *n* = 6
Muscle	0.14 ± 0.011 *n* = 3	0.10 ± 0.027 *n* = 6	0.11 ± 0.047 *n* = 5	0.17 ± 0.089 *n* = 6
Dorsal fat	0.60 ± 0.35 *n* = 6	0.28 ± 0.19 *n* = 6	0.16 ± 0.064 *n* = 4	0.11 ± 0.033 *n* = 5
s.c. fat	1.1 ± 1.1 *n* = 6	0.45 ± 0.46 *n* = 5	0.18 ± 0.086 *n* = 5	0.27 ± 0.17 *n* = 5
Perirenal fat	8.8 ± 5.6 *n* = 6	1.0 ± 1.2 *n* = 5	2.0 ± 1.7 *n* = 6	4.6 ± 3.7 *n* = 6
Bile fluid	57 ± 100 *n* = 6	37 ± 71 *n* = 6	3.7–8.6 *n* = 3^1^	7.5–120 *n* = 2^2^
Duodenum content	82 ± 72 *n* = 6	13 ± 15 *n* = 6	0.59 ± 0.48 *n* = 5	9.0 ± 17 *n* = 6

Stated concentrations approximated

*s.c.*  subcutaneous, *PB* peripheral blood, *CB* central blood, *neg.* negative, *n.a.* not available

^1^Only sampled from 3 pigs

^2^Only sampled from 2 pigs, as nor more fluid was available

These findings are in rather good accordance with those of previous studies with the 7-azaindol SC *cumyl*-5F-P7AICA (Walle et al. 2024) as well as the synthetic cannabinoids JWH-210 and RCS-4 of the older generation (Schaefer et al. 2019). Yet, one discrepancy was found concerning concentrations in lung tissue. While in those studies highest concentrations were determined in this tissue, in the present study lowest concentrations were detected in lungs. At first glance, this result appears somewhat surprising, as all synthetic cannabinoids previously investigated were administered by inhalation. Reflecting an explanation, a lower lipophilicity of 5F-MDMB-P7AICA compared to the other synthetic cannabinoids might be one reason for a negligible pulmonary first-pass uptake (Bakhle 1990; Bend et al. 1985; Boer 2003). Besides this, 5F-MDMB-P7AICA contains an ester structure in the linked group, resulting in a fast degradation to the DBA metabolite, as already reported by Krotulski et al. (2020). This might also be the reason for the generally much lower concentrations of the parent compound in the different specimens as compared to the older synthetic cannabinoids JWH-210 and RCS-4 as well as *cumyl*-5F-P7AICA. Yet, another explanation for the lower concentrations as compared to those determined by Walle et al. (2024) for *cumyl*-5F-P7AICA could be the longer duration of the experiments amounting to 8 h. In the study by Walle et al., the animals were already put to death after 6 h.

To assess whether a substance is underlying PMR, two markers are described in the literature. The central-to-peripheral blood (C/P) concentration ratio > 1, and the liver-to-peripheral blood (L/P) ratio > 5 or 20–30 indicate a PMR (Han et al. 2012; McIntyre 2014). Calculating those ratios for the present SC leading to ratios lower than 1 or 5, respectively indicates a low trend for PMR.

The DBA could be detected in every specimen except for brain. This tissue was tested negative for the metabolite. The metabolite was detected in relatively high amounts in urine, bile fluid and duodenum content samples. Rather high amounts were also found in kidney and perirenal fat samples. In the remaining tissues, rather low amounts of DBA of mostly less than 1 ng/g could be found.

Comparable findings have also been reported in previous studies using JWH-210, RCS-4 and *cumyl*-5F-P7AICA (Schaefer et al. 2020a; Walle et al. 2024). Analogously, the high concentrations in bile fluid and duodenum content suggest an enterohepatic circulation (Schaefer et al. 2017, 2019). In correspondence to the findings of the parent compound, resulting from ester cleavage, overall higher concentrations of the DBA metabolite were found especially in the specimens related to metabolism and elimination. The ester cleavage might also explain that we found higher metabolite concentrations than Walle et al. for the metabolite of *cumyl*-5F-P7AICA (Walle et al. 2024).

For comparison of the tissue distribution pattern with data from authentic fatal cases only one report on a fatal case with a contribution of 5F-MDMB-P7AICA to the occurrence of death and a comparable survival time after drug intake was available (Walle et al. 2023). In this fatal case PB and CB concentrations of 1.2 and 0.69 ng/mL were found, respectively. These concentrations are consistently around twice as high as those found in the pigs 8 h after administration. The DBA concentrations of 5.7 ng/mL in PB and 46 ng/mL in CB were considerably higher than those found in our systematic study. In the tissues and bile fluid, only the DBA metabolite could be detected with highest concentration in bile fluid. These results differ from those found in the present study examining pig tissues, in as far as the parent compound was also determined in organ tissues. Yet, single case reports are generally fraught with imponderabilities, as usually neither the consumed dose and time of consumption nor the PMI are known. However, both studies have one interesting finding in common. The fact that the concentrations of the parent in PB were mostly twice as high as those in CB.

### PM concentrations and concentration changes

The mean concentrations and SD of the parent and DBA calculated in PM specimens are listed in Table 2. The median PM concentrations are depicted in Fig. 1A and B. As the pigs were catheterized, no urine specimens could be sampled PM. Looking at the distribution in the different organs and body fluids, highest concentrations of the parent substance were observed in adipose tissue specimens sampled from different locations followed by bile fluid. Those findings are in good agreement with already investigated synthetic cannabinoids and might be explained by a higher lipophilicity (sequestration in adipose tissue) as well as an enterohepatic circulation (storage in bile fluid) (Schaefer et al. 2020a, 2020b; Walle et al. 2024). Overall, lowest concentrations were determined in kidney and brain tissue. The very low concentrations in kidney might be a result of the parent being extensively metabolized and renally excreted as DBA. The low concentrations in brain tissue are a bit astonishing, because this organ is the site of action. Yet, unpublished data of the authors indicate that 5F-MDMB-P7AICA is a substrate of the *P*-glycoprotein. As the protein is expressed in the blood–brain barrier, the transmissibility into the central nervous system of 5F-MDMB-P7AICA may be reduced.

Highest concentrations of the DBA were determined in duodenum content, bile fluid as well as liver and kidney tissue. These findings seem not to be surprising, as those organs and body fluids are involved in metabolism and excretion processes. Lowest concentrations were found in muscle, brain, and blood specimens.

In the PM specimens, in terms of absolute concentrations, only negligible changes of concentrations were observed in both parent compound and metabolite in the body fluids and tissues. Especially PB concentrations remained rather constant over the time of observation. Only in liver a slight increase of 5F-MDMB-P7AICA and 5F-MDMB-P7AICA-DBA was detected over time. Regarding the parent compound, concentrations in liver specimens at PMI 2 and 3 were significant higher (*p* < 0.05) than those at PMI 0. This increase may be explained by the anatomical vicinity to bile and duodenum. In those organs rather high concentrations were determined in specimens collected immediately after death (PMI 0), and correspondingly decreasing concentrations were observed in the PM specimens. This decrease was statistically significant (*p* < 0.05) concerning concentrations of the DBA at PMI 2 as compared to those at PMI 0. Calculating the C/P and L/P concentration ratios for 5F-MDMB-P7AICA from PMI 1–3 revealed also ratios < 1 and < 5 as for the PMI 0. In line with the rather stable absolute concentrations, these ratios further substantiate a negligible PMR-potential.

The rising concentrations of the parent compound in muscle tissue could be the result of PMR from adipose tissue. Concentrations in muscle tissue significantly increased (*p* < 0.05) from PMI 0 to PMI 3. Correspondingly, the concentrations in adipose tissue overall showed a tendency to decrease, despite a rather high interindividual deviation. This tendency could also be observed for the rather low metabolite concentrations in dorsal and subcutaneous fat specimens, resulting in significant lower concentrations over time. Differing therefrom, DBA-concentrations determined in perirenal adipose tissue showed a slight increase over the PMI. A possible explanation could be a PMR from surrounding renal tissue. Taken together, the DBA metabolite also seems not to be subject of a PMR.

One interesting result was the quite stable concentrations of 5F-MDMB-P7AICA in CB over the experimental time of 72 h. Hence, CB also seems to be suitable for a PM quantification, when no PB can be obtained. To sum up the results of our study, bile fluid/duodenum content, muscle and kidney tissue as well as adipose tissue seem to be appropriate specimens for a qualitative PM detection of a consumption of 5F-MDMB-P7AICA. In the case that PB is not available for quantification, CB seems to be a suitable alternative specimen, since no PMR was observed in the present study.

### Limitations

The most important limitation of the study is the permanent re-opening of the abdominal cavity to collect PM specimens. This repeated opening may lead to a more pronounced contamination with microorganisms and aerobic conditions inside the body. As a result, a faster putrefaction might occur. In addition, specimens were sampled from different sites of the organs, possibly affecting the concentrations found, if the analytes were not homogenously distributed. The PB was collected from different vessels, as blood coagulated inside the vessels or diffused postmortem to lower regions of the body. So, we were not able to sample enough volume from only one vein for the whole period. This could also have an influence on the concentrations of the analytes.

## Conclusions

In the present study, the perimortem distribution patterns of 5F-MDMB-P7AICA and its DBA metabolite following inhalative administration to pigs was assessed. Subsequently, the PM distribution patterns as well as possible time-dependent concentration changes were determined. In general, both substances were distributed all over the body except for brain and kidney tissue. In the latter, the parent was not found, whereas in brain tissue, the metabolite was not present right after death. Unlike other substances, CB seems to be an alternative matrix for reliable quantification. If no standard specimens, such as PB and CB, are available, bile fluid/duodenum content, muscle and kidney as well as adipose tissue are useful for qualitative PM analysis. Overall, no relevant PMR was observed for both 5F-MDMB-P7AICA and its DBA metabolite.

## Supplementary Information

Below is the link to the electronic supplementary material.

Supplementary file1 (DOCX 17 KB)

## Data Availability

The data that support the findings of this study are available from the corresponding author upon reasonable request.
