# Supplementary material for: Does a postmortem redistribution affect the concentrations of the 7 azaindole-derived synthetic cannabinoid 5F-MDMB-P7AICA in tissues and body fluids following pulmonary administration to pigs?
Source: Arch Toxicol. 2024 Jul 2;98(10):3289–98. doi: 10.1007/s00204-024-03815-1 (PMC11402846; doi:10.1007/s00204-024-03815-1)
Supplement: Supplementary file 1 — Supplementary file1 (DOCX 17 KB) [file 204_2024_3815_MOESM1_ESM.docx]

**Electronic Supplementary Material for Archives of Toxicology**

**Does a postmortem redistribution affect the concentrations of the 7 Azaindole derived synthetic cannabinoid 5F-MDMB-P7AICA in tissues and body fluids following pulmonary administration to pigs?**

Adrian A. Doerr, Frederike Nordmeier, Nadja Walle, Matthias W. Laschke, Michael D. Menger, Markus R. Meyer, Peter H. Schmidt and Nadine Schaefer^*^

Materials and Methods

**Supplementary Table 1:** Molecular formula, CAS number, SMILES ID and InChi code of 5F-MDMB-P7AICA, 5F-MDMB-P7AICA-dimethyl butanoic acid metabolite and AB-FUBINACA-d4 each.

|  | **5F-MDMB-P7AICA** | **5F-MDMB-P7AICA-dimethyl butanoic acid metabolite** | **AB-FUBINACA-d_4_** |
| --- | --- | --- | --- |
| **Molecular formula** | C_20_H_28_FN_3_O_3_ | C_19_H_26_FN_3_O_3_ | C_20_H_17_D_4_FN_4_O_2_ |
| **CAS number** | 2377403-81-9 | 2712863-53-9 | 2747917-48-0 |
| **SMILES ID** | O=C(N[C@H](C(OC)=O)C(C)(C)C)C1=CN(CCCCCF)C2=C1C=CC=N2 | O=C(N[C@H](C(O)=O)C(C)(C)C)C1=CN(CCCCCF)C2=C1C=CC=N2 | O=C(N[C@H](C(N)=O)C(C)C)C1=NN(CC2=CC=C(F)C=C2)C3=C1C([2H])=C([2H])C([2H])=C3[2H] |
| **InChi code** | InChI=1S/C20H28FN3O3/c1-20(2,3)16(19(26)27-4)23-18(25)15-13-24(12-7-5-6-10-21)17-14(15)9-8-11-22-17/h8-9,11,13,16H,5-7,10,12H2,1-4H3,(H,23,25)/t16-/m1/s1 | InChI=1S/C19H26FN3O3/c1-19(2,3)15(18(25)26)22-17(24)14-12-23(11-6-4-5-9-20)16-13(14)8-7-10-21-16/h7-8,10,12,15H,4-6,9,11H2,1-3H3,(H,22,24)(H,25,26)/t15-/m1/s1 | InChI=1S/C20H21FN4O2/c1-12(2)17(19(22)26)23-20(27)18-15-5-3-4-6-16(15)25(24-18)11-13-7-9-14(21)10-8-13/h3-10,12,17H,11H2,1-2H3,(H2,22,26)(H,23,27)/t17-/m0/s1/i3D,4D,5D,6D |

Surgical Procedures

The premedication was administered by intramuscular injection and consisted of ketamine hydrochloride (30 mg/kg, Ursotamin; Serumwerk Bernburg, Bernburg, Germany), xylazine hydrochloride (2.5 mg/kg, Rompun; Bayer, Leverkusen, Germany) and atropine (1 mg, Braun, Melsungen, Germany). Isoflurane (2‑4 %, Forene, AbbVie Ludwigshafen) was used to maintain analgosedation. Volume cycled ventilation was conducted with a mixture of oxygen and air (1:2 v/v; FiO2 of 0.30; Respirator ABV-U; F. Stephan GmbH, Gackenbach, Germany) and a tidal volume of 10 to 12 mL/kg. Body fluids were replaced by infundation of a 0.9% sodium chloride solution (8 mL per kg BW per hour; Braun, Melsungen, Germany) via the left ear vein. To monitor the mean central venous pressure, a triple-lumen 7F (Certofix Trio, Braun, Melsungen, Germany) central venous catheter was inserted into the jugular vein. Additionally, a cathether was placed into the femoral arteria to measure the arterial blood pressure. Heart rate was monitored by electrocardiography. Pulse and oxygen saturation were determined by an oximeter placed around the tongue. Subsequently, the animals were allowed to stabilize for 10-15 min.

Apparatus

A Thermo Fisher (TF, Dreieich, Germany) HPLC consisting of one Allegro pump and an HTC PAL autosampler coupled to a TF TSQ Quantum Ultra Accurate Mass triple stage mass spectrometer with an electrospray ionization (ESI) interface run in positive mode was used.

The settings of the mass spectrometer were as follows: vaporizer temperature, 380 °C; capillary temperature, 290 °C; collision cell pressure, 1.5 mTorr; sheath gas, 40 arbitrary units (AU); ion sweep gas, 5 AU; auxiliary gas, 20 AU; spray voltage, 4.000 V. Elution was performed by using a gradient of mobile phases A and B. For gradient elution, a Macherey-Nagel (Düren, Germany) C18 endcapped column (150 x 2 mm, 5 μm) was applied using mobile phase A and B. The flow rate was set to 0.5 mL/min. The gradients used started from 0‑1 min with 25 % of solvent B, increased from 1 to 5 min to 100 % of solvent B, which was kept from 5‑9 min. After that, the starting conditions were restored and kept for 1 min. The total runtime was set to 10 min. The detection of the compounds was performed in the multiple-reaction monitoring mode with three transitions per precursor ion. For evaluation, the TF Xcalibur Version 2.0.7 SP 1 software was used. The transitions are listed in Supplementary Table 1.

**Supplementary Table 2:** Instrumental conditions: Precursor ion, product ions, collision energies and scan time of 5F-MDMB-P7AICA, 5F-MDMB-P7AICA-dimethyl butanoic acid metabolite and AB-FUBINACA-d_4_ each. Underlined product ion used as target.

|  | **Precursor ion [m/z]** | **Product ion [m/z]** | **Collision energy [eV]** | **Scan time [ms]** |
| --- | --- | --- | --- | --- |
| **5F-MDMB-P7AICA** | 378.00 | 145.01 233.02 116.96 | 38 20 53 | 50 50 50 |
| **5F-MDMB-P7AICA-dimethyl butanoic acid metabolite** | 364.10 | 145.14 233.18 117.21 | 35 18 59 | 50 50 50 |
| **AB-FUBINACA-d_4_** | 373.20 | 257.18 328.29 | 24 15 | 50 50 |

**Supplementary Table 3:** Vital parameters at the time of death: blood pressure, pulse, rectal temperature and O_2_-saturation.

|  | Blood pressure [mmHg] | Pulse [1/min] | Rectal temperature [°C] | O_2_-saturation [%] |
| --- | --- | --- | --- | --- |
| Pig 1 | 81/40 | 74 | 37.2 | 94 |
| Pig 2 | 80/41 | 75 | 38.2 | 85 |
| Pig 3 | 72/40 | 73 | 37.6 | 93 |
| Pig 4 | 74/36 | 91 | 38.8 | 93 |
| Pig 5 | 64/24 | 102 | 39.6 | 89 |
| Pig 6 | 72/30 | 75 | 37.1 | 86 |
